# Structures, Biological Activities and Phylogenetic Relationships of Terpenoids from Marine Ciliates of the Genus *Euplotes*

**DOI:** 10.3390/md8072080

**Published:** 2010-07-08

**Authors:** Graziano Guella, Danielle Skropeta, Graziano Di Giuseppe, Fernando Dini

**Affiliations:** 1 Bioorganic Chemistry Lab, Department of Physics, University of Trento, 38050 Povo, Trento, Italy; 2 CNR, Istituto di Biofisica Trento, Via alla Cascata 56/C, 38050 Povo, Trento, Italy; 3 Unit of Protistology, Department of Biology, University of Pisa, 56126 Pisa, Italy; E-Mails: gdigiuseppe@biologia.unipi.it (G.D.G.); fdini@biologia.unipi.it (F.D.)

**Keywords:** marine ciliates, terpenoids, biosynthesis, bioactivity, phylogenetic relationships

## Abstract

In the last two decades, large scale axenic cell cultures of the marine species comprising the family Euplotidae have resulted in the isolation of several new classes of terpenoids with unprecedented carbon skeletons including the (i) euplotins, highly strained acetylated sesquiterpene hemiacetals; (ii) raikovenals, built on the bicyclo[3.2.0]heptane ring system; (iii) rarisetenolides and focardins containing an octahydroazulene moiety; and (iv) vannusals, with a unique C_30_ backbone. Their complex structures have been elucidated through a combination of nuclear magnetic resonance spectroscopy, mass spectrometry, molecular mechanics and quantum chemical calculations. Despite the limited number of biosynthetic experiments having been performed, the large diversity of ciliate terpenoids has facilitated the proposal of biosynthetic pathways whereby they are produced from classical linear precursors. Herein, the similarities and differences emerging from the comparison of the classical chemotaxonomy approach based on secondary metabolites, with species phylogenesis based on genetic descriptors (SSU-rDNA), will be discussed. Results on the interesting ecological and biological properties of ciliate terpenoids are also reported.

## 1. Introduction

Ciliate is the common name assigned to a protist taxon comprising the phylum Ciliophora. Over 7000 ciliate species have been described from marine, freshwater and terrestrial habitats, where they play a key role in microbial food webs. These eukaryotic microorganisms are characterized by a nuclear dualism and body-covering cilia used for locomotion and feeding. Relying solely on their outwardly visible characteristics as the main discriminating parameters for identifying protistan species, some claim that protists show a poor biodiversity [1,2] and thereby wrongly assume that this is accompanied by poor chemical diversity [3]. However, there is increasing evidence that protists express a biodiversity comparable to other taxa [4,5]. Furthermore, the genetic distance among ciliate taxa of the same taxonomic rank, species included, is far larger than that occurring among many multicellular representatives. The fact that protists violate the first species property (*i.e.*, morphological uniqueness) does not imply that other species-specific properties cannot undergo selective pressures leading to new, separate evolutionary units. The low level of biodiversity found in protists to-date is likely to be a reflection of the lack of workable methods for addressing the species problem in protists. Hence, it may frequently occur that two strains showing the same identifying, morphological features behave quite differently. Molecular genetic methodologies have provided a powerful means to address the protistan systematic-taxonomic problems. The outcomes of these new approaches strongly suggest that establishment of protistan biodiversity is a task that has just commenced [6]. Moreover, in the last two decades detailed investigation of ciliate secondary metabolites from the genus *Euplotes* has added “a new dimension” to the problem of their species-specific allocation via a “chemotaxonomic” approach, which is able to define protistan taxonomy to the subspecific level [7]. In line with current views of protistan microbiologists, the results obtained by the latter approach have highlighted a rich diversity of ciliate secondary metabolites. Further investigation into the morphological, physiological, and ecological properties of this interesting taxon is therefore essential, especially given the interest in their exploitation as new biotechnological tools.

**Scheme 1:**
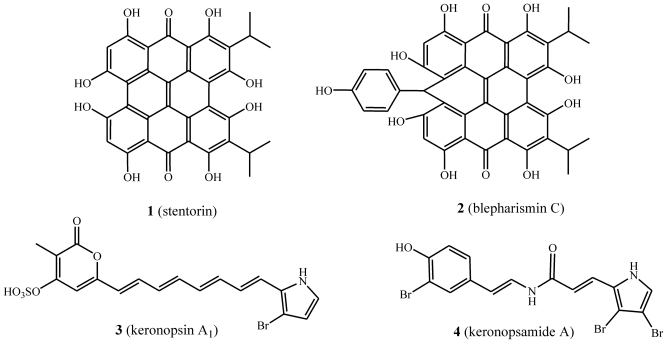
Pigments isolated from freshwater and marine ciliates.

From a natural products point of view, ciliates are microorganisms able to synthesize, *inter alia*, secondary metabolites belonging to three main biogenetic classes. The first class is represented by pheromone peptides and proteins, of which structural, functional, and evolutionary properties have been described by Luporini and co-workers. These cell-specific signal proteins, isolated from ciliated protozoa belonging to the genus *Euplotes*, constitutively diffuse into the extracellular environment [8]. Among the several peptide pheromones isolated from marine ciliates, the shortest show sequences of 38–40 amino acids arranged to form a bundle of three α-helices, which have an up-down-up orientation and are maintained in close juxtaposition by three to four disulfide bonds located in conserved positions within the family. From a functional point of view, these peptides have been demonstrated to induce mating of cells via paracrine-like (or heterotypic) interactions and, as a consequence of their autocrine binding, to promote the vegetative (mitotic) proliferation of the same cells from which they are released. The second class of products reported from ciliates is broadly defined as ciliate pigments, which are compounds of mixed biosynthetic origin. In particular, ciliates are known to produce three main families of pigments (Scheme 1). The first contains metabolites built on the hypericin skeleton such as stentorin (**1**) [9] and blepharismin C (**2**) [10,11]; the second is represented by compounds wherein a ß-bromine substituted pyrrole is linked to a sulfate pyrone through an extended conjugated chain, such as keronopsin (**3**) [12]; and the last derives from a peptide-like linked brominated tyramine with a brominated pyrrole carboxylic acid, such as the keronopsamides A (**4**) [13]. Although the bioactive alkyl-resorcinols isolated from *Climacostomum virens* [14], the so called “climacostol family”, are not true pigments, they can broadly be thought to belong to this class, being metabolites of mixed biosynthetic origin.

From the early 90s, investigation into the natural products from marine ciliates belonging to the genus *Euplotes* revealed a dominance in the production of terpenoids. At that time, only one study on crude extracts of *Tetrahymena pyriformis* reported the production of the polycyclic triterpene tetrahymanol, an important metabolite along the squalene cyclization cascade [15]. In 1992, the ability to obtain large mass cultures of the marine morphospecies *Euplotes crassus* led to the isolation of the highly strained acetylated hemiacetals, the first sesquiterpenoids from marine protists. Since then, the intra- and interspecific diversity of ciliates has been widely investigated.

**Figure 1:**
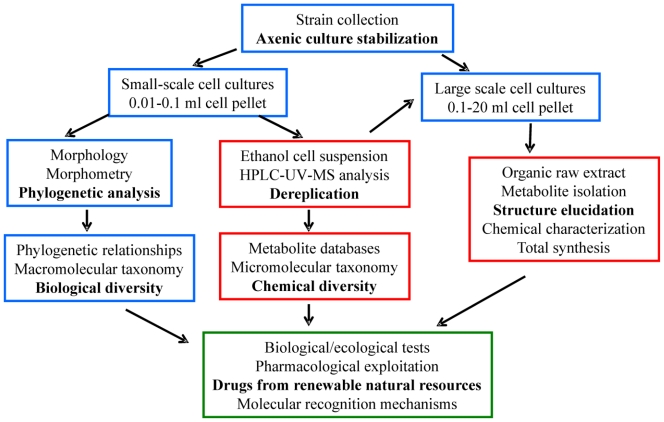
The biological (blue boxes) and chemical (red boxes) steps of an integrated approach, leading to an understanding of the functional roles of secondary metabolites from marine ciliates (green boxes).

This review summarizes the results of almost two decades of research in the field by the authors, but also, and more importantly, presents a unifying picture of the chemical and biological results, including both reported and previously unpublished data. The review begins with a short description of the approach used to tackle the topic. As shown schematically in Figure 1, a general methodology that relies on strong collaboration between microbiologists, molecular biologists and natural product chemists is essential. For each investigated morphospecies the following is discussed: (a) the chemical structures and reactivity of the isolated terpenoids; (b) the intra-species chemical diversity; (c) putative biosynthetic pathways accounting for the formation of the isolated terpenoids; (d) the intra-species biological diversity through phylogenetic relationships; and (e) the results of bioactivity screening. The evolutionary significance of these metabolites is described in the last section, along with a comparison of the outcomes from the analysis of micromolecular markers (terpenoids) with that of macromolecular descriptors (SSU-rRNA).

## 2. Generalities on Biological and Chemical Methodologies

### 2.1. Cell cultures, ecological tests and phylogenetic analysis

All the *Euplotes* strains collected were established starting from single naturally occurring cells to obtain cellular line clones, which were fed on both microalgae and bacteria. Mass cultures were grown in salt water (32‰ salinity), sterilized, and inoculated with microalgae, with the latter first incubated at 23 ± 1 °C in a 12 h light/dark cycle for at least 10 days using a daylight (Osram Daylight lamp, 36 W/10) and fluorescent (Osram Fluora lamp, 40 W/77) illuminated incubator system. Ciliates thrive in a continuously percolating culture of the autonomously reproducing autotrophic microalgae, representing a renewable source.

Starved cells from mass cultures of the strains were pelletted by successive centrifugation rounds to provide from 0.01–0.1 mL of closely packed ciliates for small-scale cell cultures to 0.1–20 mL for large-scale samples. Small-scale cell cultures were used for the morphological and phylogenetic analyses. Morphological information was obtained from living and fixed specimens, employing differential interference contrast microscopy (DIC) and scanning electron microscopy (SEM). The silver nitrate impregnation protocol devised by Foissner *et al.* [16] and the standard Feulgen staining procedure were applied after cell fixation in Bouin’s fluid to reveal the nuclear apparatus. In assigning strains to the appropriate morphospecies, the intra- (within and between strains) and inter-population variability was considered. Eleven characters commonly used for morphological distinctness among *Euplotes* taxa were chosen. The determination of the phenotypic traits was carried out on 10 selected specimens of each preparation at 1000X magnification on a Leica DMR microscope, using the dedicated Leica IM1000 version 1.0 software STATISTICA (StatSoft, Inc., USA). Data produced by this morphometric procedure were analyzed using the multivariate technique of discriminating functions.

To gain insight into the taxonomic and phylogenetic relationships among congeneric species of *Euplotes*, the nuclear small subunit rRNA (SSU-rRNA) gene was selected to determine the phylogenetic trees. The nuclear SSU-rRNA gene sequences were amplified by polymerase chain reaction (PCR) of DNA prepared from small-scale cell cultures concentrated by centrifugation and incubated in lysis buffer (0.5 M EDTA, 1% SDS, 10 mM Tris-HCl, pH 9.5) containing proteinase K (200 μg/mL), at 55 °C for 12–15 h. The universal eukaryotic forward primer 18S F9 (5′-CTGGTTGATCCTGCCAG-3′) [17] and the 18S R1513 Hypo reverse primer (5′-TGATCCTTCYGCAGGTTC-3′) were used [18]. The PCR products were directly sequenced in both directions with one forward [F783 (5′-GACGAAATCAAAGAAATACCGTC-3′)] and two reverse [R536 (5′-CTGGAATTACCGCCGGCTG-3′) and R1052 (5′-AACCTTAAGGAACCCCCG GCCATGGCAA-3′)] internal primers [18]. The SSU-rRNA gene sequences were aligned using the program ClustalX [19] with default parameter settings. The alignment was adjusted interactively with the program BioEdit Sequence Alignment Editor [20] to optimize the base-pairing scheme of the rRNA gene molecule secondary structure [21]. The phylogenetic analysis was performed in PAUP version 4.0b10 [22], applying a neighbor joining method and using Modeltest 3.06 [23] to select the appropriate model of substitution. The Akaike Information Criterion (AIC) indicated that the Tamura-Nei model [24], which considers unequal base frequencies, was the most appropriate. The reliability of the internal branches of the phylogenetic tree was assessed using the bootstrap method [25].

Biological/ecological tests were carried out on various strains of the different morphospecies. Responses of strains to metabolite treatments were assessed by examining the impact on cell fission rates and cell tolerance. Cleaving cells were isolated from well-fed cultures, thus producing groups of physiologically synchronized single progeny individuals. These fission progeny cells normally underwent at least one fission in ciliates of larger size, or two fissions in ciliates of smaller size. Using a micropipette, they were individually transferred to wells of three-spot hemispheric depression slides for 16 h incubation in salt water, sterilized, and then assessed in the absence (control) or in the presence of metabolite. The cultures were kept under observation by light microscopy. The effects of each experimental treatment, as well as the controls, were scored on a series of nine cells, individually distributed into the depressions. The fission rate of a cell’s progeny was calculated using the formula: n = log_2_ n_x_; where n is the fission number performed by a single cell since its isolation into a depression, and n_x_ is the number of cells scored after 16 h. Cells that were unable to swim or creep on the bottom of the depression, or with motionless cirri (or both), were considered dead.

All the terpenoids reported here, including those isolated from *E. focardii*, have been tested using an unbiased sample of marine interstitial ciliates in order to verify their biological activities.

### 2.2. Cell extracts, de-replication analysis, isolation and structure elucidation of secondary metabolites

Since secondary metabolites in ciliates are not-dispersed in the culture-medium, cells obtained by centrifugation were extracted in ethanol and stored in the same solvent (at -20 °C) prior to analysis. Rapid identification and quantitative estimates of known compounds were obtained from the ethanol extracts using de-replication techniques based on liquid chromatography-tandem mass spectrometry (LC-MS/MS) methods. Photo diode array was used for UV-Vis detection, while sample MS ionization used an ESI or APCI ion-source (in either positive- or negative-ion mode) coupled to an ion trap mass analyzer. An aliquot of 5 μL of the ethanol solution of each sample (the supernatant of the cell suspension containing about 0.2 mL of cell-pellet in 1 mL of alcohol) was injected into a C18 column (4.6 × 150 mm, 3.5 μm) with a 7:3 mixture of CH_3_CN/H_2_O as the eluent and a flow rate of 0.9 mL/min.

In order to isolate pure metabolites, the ethanol solution obtained through cell pellet filtration was evaporated and the residue partitioned between hexane-ethyl acetate 9:1 (organic part) and methanol-water 9:1 (aqueous part). The organic extract was then subjected to reversed-phase flash chromatography (RP-FC) on a Lichrolut RP18 column with a CH_3_CN-MeOH gradient elution, collecting 5 fractions of about 2 mL. The first fractions that eluted usually contained the terpenoid metabolites, which could be further purified by RP semi-preparative HPLC (RP18, CH_3_CN-H_2_O 7:3, 5 mL/min). The latter FC fractions contained different classes of lipids such as sterols, di- and triacylglycerols, free fatty acids and phospholipids. Structural elucidation of the compounds under investigation was performed using nuclear magnetic resonance (NMR) spectroscopy, mass spectrometry (MS) and infrared spectroscopy (IR), combined with the results from molecular mechanics (MMX and MM3 force fields) and density functional theory (DFT).

## 3. Terpenoids from *Euplotes crassus*

### 3.1. Structures, chemical reactivity and total synthesis

**Figure 2:**
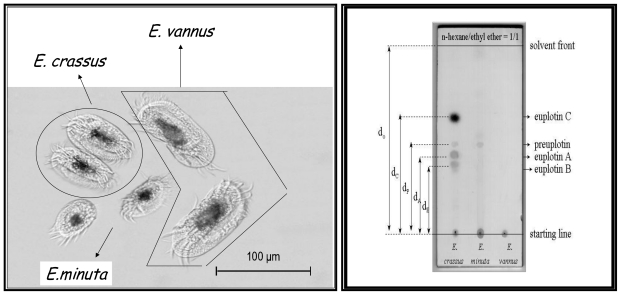
Left: Transmission electron microscopy (TEM) image of *Euplotes crassus*, *Euplotes minuta* and *Euplotes vannus* ciliates; Right: TLC plate (stationary phase: Merck Si60 PF_254_, eluent: n-hexane-ethyl ether 1:1, staining reagent: Ce(SO_4_)_2_/H_2_SO_4_) of crude cell extracts (0.1 mL of cell pellet) from typical strains of *E. crassus*, *E. minuta* and *E. vannus.*

When investigation into marine ciliated protozoa in the subclass Hypotrichida first began, morphological data proved ambiguous in deciding whether *Euplotes vannus* and *Euplotes crassus* were separate species or part of a species complex. Indeed, the *Euplotes vannus-crassus-minuta* group is a complex of closely related, cosmopolitan, marine ciliates, for which the ability to distinguish between them continues to be contentious. Three morphospecies have been identified with *E. vannus* being the largest form, *E. minuta* the smallest, and *E. crassus* presenting intermediate morphological characteristics. In particular, the morpho-variability of *E. vannus* and *E. crassus* is low and their microscopic identifying features are not clearly separated (Figure 2, left).

**Scheme 2:**
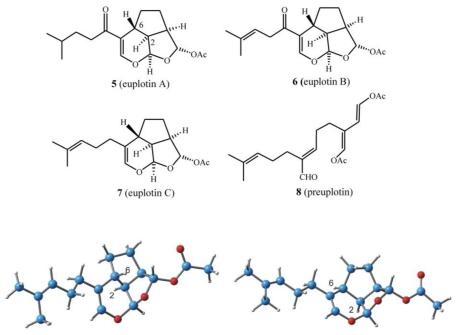
Top: Sesquiterpenoids **5**–**8** isolated from cell cultures of *Euplotes crassus*. Bottom: Molecular mechanics minimized structures of euplotin C (left, *trans* 2–6 ring junction) and of its hypothetical more stable C(6) epimer (right, *cis* 2–6 ring junction).

Starting with the most frequently sampled hypotrichid *E. crassus*, strains collected in different geographical sites have all been found to give the same secondary metabolites, referred to as the euplotins (Scheme 2). These are sometimes also accompanied by their putative acyclic precursor, preuplotin [26]. Thin-layer chromatography (TLC) can be used to distinguish the protozoan *E. crassus* as it is the only euplotin producer within the *E. vannus-crassus-minuta* complex of sibling species. As shown in Figure 2, for *E. crassus* extracts, three UV active spots ascribed to euplotin A, B and preuplotin are detected, along with a dark spot (Retention factor, R_f_ 0.62) for euplotin C, which appears upon staining. For *E. minuta*, only preuplotin is detected, while strains of the *E. vannus* morphospecies contain neither euplotin nor preuplotin.

**Figure 3:**
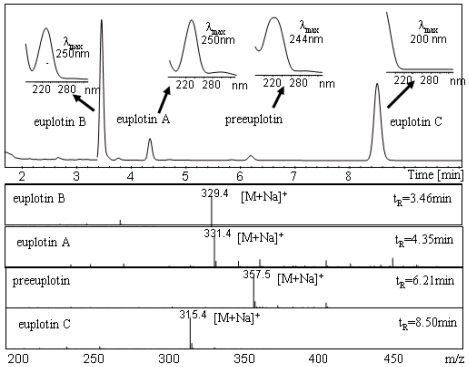
LC-UV chromatogram λ 220 nm) of a crude extract of *Euplotes crassus* (strain Pdr1): for all metabolites UV spectra are reported on the top and ESI(+)-MS spectra at the bottom.

A typical chromatogram obtained from analysis of an *E. crassus* strain (Pdr1) is reported in Figure 3, showing the chromatographic separation of the euplotins, characterized by peak-selective UV and ESI-MS ion spectra. Depending on the environment of the electrospray ionization chamber, concentration and composition of the eluate, the sodium adduct [M + Na]^+^ of the molecular ion is usually detected in the positive-ion mode ESI-MS spectrum, followed by potassium adducts [M + K]^+^ and/or dimeric ion-species [2M + 2Na]^2+^. This analysis provides both qualitative and quantitative data and is currently employed in the initial de-replication analysis of new strains. With a single HPLC-UV-MS run, three important pieces of molecular information are obtained simultaneously: polarity (retention time, t_R_), chromophoric groups (UV-Vis spectra), and molecular weight (MS spectra) of every metabolite eluted by the LC system. These data can also be used to track metabolic changes during the life-cycles of the microorganisms.

In all investigations on different strains of *E. crassus*, the acetylated sesquiterpenoid euplotin C (**7**) was the most abundant metabolite, and can thus be used as a chemotaxonomic marker of this morphospecies. Euplotin C is often followed by at least one of its structural analogs, euplotin A (**5**) or euplotin B (**6**), and occasionally by the putative acyclic precursor, preuplotin (**8**) (Scheme 2) [27].

The euplotane skeleton is a tricyclic ring system, built on a trialdehyde-masked system wherein an unusual 2,6-*trans*-stereochemistry imposes a high degree of strain on the overall structure. According to *ab initio* DFT calculations, the 2,6-*trans* ring junction leads to an increase in the euplotin strain energy of about 12 kcal/mol compared to that of the diastereoisomeric 2,6-*cis* analog (Scheme 2, bottom). Interestingly, the structural features of the latter are present in udoteatrial hydrate [28], a diterpenoid isolated from the marine alga *Udotea flabellum*.

**Scheme 3:**
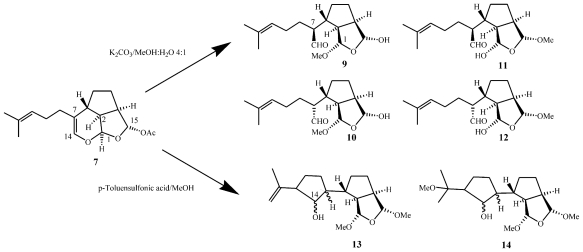
Hydrolysis of euplotin C (**7**) under either mildly basic or acidic conditions.

Another feature of the euplotins is their high chemical instability [16]. In mild basic conditions, euplotin C readily undergoes a cascade of irreversible transformations, leading to four major products, as regio- and diastereoisomeric mixtures (**9**–**12** in Scheme 3). Loss of the acetate group at C(15), releases the strain imposed by the *trans* 2–6 ring junction in the euplotane skeleton, since the incipient C(14) aldehyde in the hypothetical trialdehyde intermediate is far from the C(1) aldehyde group, regardless of the relative stereochemistry. Curiously, while C(1) and C(15) are strongly involved in mutual acetal condensations induced by nucleophilic attack of MeOH, the C(14) aldehyde group does not cause any acetalization. The analysis of the end-products of this reaction confirmed the euplotins structure, and also allowed the establishment of their absolute configuration through the application of the modified Mosher method [30,31] involving MTPA esterification of the hemiacetal groups of the regioisomers **9** and **10** [16].

Different results were obtained when euplotin C (**7**) was subjected to mildly acidic conditions (e.g., *p*-toluensulfonic acid in methanol). NMR analysis of the reaction products indicated complete acetalization at C(1) and C(15) (no hemiacetals present) and, more importantly, that the incipient C(14) aldehyde group was trapped by the sp^2^ C(12) in a carbonyl-ene type reaction, affording the expected hydroxy-cyclopentane ring [as an epimeric mixture at C(14)] as in the derivatives **13** and **14**. The latter derivative, **14**, is derived from acid-catalyzed electrophilic attack of MeOH on the *iso*-propylidene moiety of **13**. As it will become clearer further on, the possibility of acid-catalyzed carbonyl-ene processes is of paramount importance since this kind of intramolecular cyclization seems to play a dominant role in the biosynthesis of the majority of terpenoids isolated from marine ciliates.

Euplotins are also amphiphilic molecules, with a “water-like” polar part defined by an unusual sequence of electron-withdrawing oxygen atoms, as well as a hydrophobic tail. It is therefore not surprising that they give rise to micellar aggregates in water, at least above their critical concentration (CMC). Although the CMC value of euplotins in aqueous solution or the size of their aggregates has not been measured, evidence of micellar phases can be obtained from line-width analysis of ^1^H-NMR spectra taken on aqueous samples containing euplotins at different concentrations. In particular, NMR experiments carried out on euplotin C (**7**) supported the formation of large molecular aggregates by the appearance of strong signal broadening in aqueous ^1^H-NMR spectra due to significant decreasing of their transversal relaxation times T_2_. These aggregates are only partially destroyed by extensive sonication treatment, as evidenced by NMR spectra signal re-sharpening.

Euplotins A–C (**5**–**7**) are even unstable in water, but the end-products are different from those discussed above in methanol solutions. In general terms, the increase of the reactant concentration in the micellar phase is expected to lead to a considerable acceleration of the hydrolysis reaction due to the well known micellar catalysis effect [31]. With euplotins as reactants, however, it could be demonstrated through LC-UV-MS techniques and deuterium incorporation experiments that three consecutive chemical processes occur. First, there is an initial fast process leading to hydrolytic cleavage of the C(15) acetate. Second, there is an electrophilic addition of water to the activated C(7)=C(14) vinyl ether double bond. Third, there is an electrophilic addition of water, catalyzed by acetic acid (deriving from process 1), to the C(10)=C(11) trisubstituted double bond of the prenyl side-chain. While the first two reactions proceed with similar rate constants, the last is much slower and becomes significant only after complete disappearance of the starting reactant, euplotin C (**7**). Free aldehydes are expected to be labile intermediates playing an equilibration role among the hemiacetal intermediates, but in these experiments they were undetectable.

Only one report has so far been published on the total synthesis of euplotins. Euplotin A (**5**, Scheme 2) has been synthesized [33] by exploiting the thermal reactivity of suitable heterodienes (2-acylalkenals) that undergo cycloaddition reactions giving the key 5-acyldihydropyran intermediate with the correct euplotane *trans* 2–6 stereochemistry. Racemic euplotin A has been obtained by functional group transformations.

### 3.2. Inclusion complexes

Since euplotins are rather hydrophobic compounds, their low water solubility represents a serious limitation to the reliability of any bioassay method. In order to overcome these difficulties, inclusion complexes of euplotin C with cyclodextrins (CD) were prepared [34]. After various trials with different CD derivatives, heptakis(2,6-di-*O*-methyl)-β-cyclodextrin (DIMEB) was finally chosen because of its binding capability and enhanced water solubility. The complex stoichiometry, binding constants and three dimensional structure of the 1:1 inclusion complex of euplotin C(**7**)-DIMEB were determined using 1D/2D-NMR techniques and LC-ESI tandem MS measurements. The data suggests that, in aqueous solution, **7**-DIMEB has a 1:1 stoichiometry, although a 1:2 ratio cannot be ruled out at least at high DIMEB/**7** molar ratio. By using LC(PDA)-MS/MS as the probe technique, kinetic investigations of the hydrolysis of the 7-DIMEB complex demonstrated that *complexed*-**7** gives rise to the same end-products as *free*-**7** alone, although every step of the overall hydrolytic process occurs at a lower rate. Thus, the increased solubility and the lower hydrolysis rate of *complexed*-**7** with respect to *free*-**7**, suggested that these inclusion complexes can be used in biological tests as reliable sources of euplotin C (**7**), avoiding the use of organic solvents such as DMSO as the carrier agent.

### 3.3. Biogenetic considerations

**Scheme 4:**
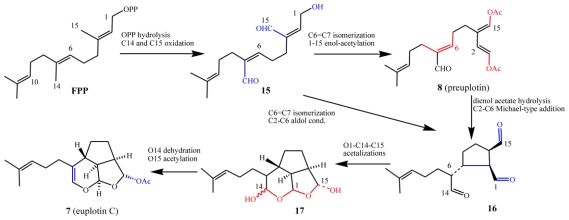
Biogenetic proposal of *Euplotes crassus* sesquiterpenoids.

A putative biosynthetic route to the euplotins from farnesyl pyrophosphate (FPP) via preuplotin (**8**) as a key intermediate is shown in Scheme 4. After C1 pyrophosphate hydrolysis, it is proposed that intermediate **15** could undergo C6=C7 double bond isomerization, along with acetylation of the OH groups at C(1) and C(15), to give the 1,4-dienol acetate moiety of preuplotin (**8**). Hydrolysis of **8** followed by nucleophilic addition of the electron rich C(2) to the electron poor C(6) would then afford the trialdehyde terpenoid **16**. Thereafter nucleophilic attack by water at C(14), followed by a cascade of internal acetalizations, would lead to euplotin C (**7**) via ß-elimination of water and O-C(15) acetylation of the hemiacetal diastereoisomeric mixture **17**. Biosynthetic experiments with labeled isotopic precursors are planned by the authors of this review, in order to support the proposed biosynthetic route described above and to establish whether or not terpenoid biosynthesis in marine ciliates follows the classical mevalonate pathway [35]. It should be noted, however, that no biogenetic data on the euplotins is currently available.

Three main aspects of the biosynthetic proposal must be highlighted because they are atypical of classical terpenoid biosynthesis. The first is that intramolecular cyclizations are not usually driven by nucleophilic additions but by carbocation-mediated, electrophilic reactions [36,37]. In fact, these cyclizations are typically initiated by a regiochemical addition of an electrophile to a sp^2^ hybridized carbon generating a carbocation, which in turn undergoes subsequent ring forming reactions. In marine organisms this process is often initiated by bromine induced electrophilic attack of double bonds leading to a plethora of haloterpenoids [38]. The second point is that in terpenoid biosynthesis, C(1) usually plays a pivotal role as the electrophilic site of the first intermediate after allylic diphosphate hydrolysis, whilst the C(14) and C(15) methyl groups are rarely used in ring forming processes. The opposite is proposed to occur in the *E. crassus* metabolism, where the key biogenetic step would be the stereoselective C(2)–C(6) bond formation, possibly deriving from conjugate addition of electron rich C(2) to the electrophilic site at C(6). In fact, the oxidation of C(14) to -CHO inverts the polarity of the C(6)=C(7) double bond making the β-carbon at C(6) particularly electron-poor. At the same time, the hydrolysis/oxidation of C(1) to -CHO makes the α-carbon C(2) an electron-rich, nucleophilic site. Finally, in classical terpenoid biogenesis, oxidations are usually thought of as final processes (sometimes even occurring as isolation artefacts), whereby the end-products (or late carbocationic intermediates) are ultimately quenched by the capture of an external nucleophile such as water.

Enzymes such as terpene cyclases that would be responsible for converting the sesquiterpene precursor FPP into species-specific secondary metabolites in ciliates are expected to be structurally and functionally different from classical cyclases involved in terpenoid biosynthesis of other phyla. This is because they are expected to recognize oxidized FPPs as substrates. Marine ciliates could, however, share the enzymatic pool leading from mevalonic acid (MVA), or from 1-deoxyxylulose-5-phosphate (DXP) [37] to isopentenyl diphosphate (IPP) and FPP.

### 3.4. Intra-morphospecies phylogenetic analysis

**Figure 4:**
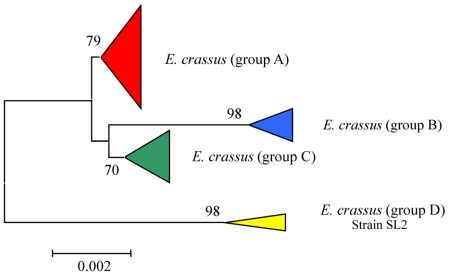
Schematic representation of the phylogenetic tree, based on SSU-rRNA gene sequence comparisons, of different strains of *Euplotes crassus*. The numbers at the nodes are bootstrap percentages from 1000 replicates. The scale bars correspond to a distance of 2 substitutions per 1000 nucleotide positions.

*Euplotes crassus* is a cosmopolitan and highly diffused marine morphospecies, with an ability to cope with changing environmental conditions [4,39,40]. This may play an important role in ecological significance of this morphospecies, while the production of the euplotins could be another element in their survival strategy [27]. The branching of the phylogenetic tree denotes, within the morphospecies analyzed, the presence of four distinct groups, all including representatives of cosmopolitan origin (Figure 4). Inter-population variation in genetic determinants following geographical separation would be expected to occur, and would result in the production of distinct secondary metabolites.

Although most strains of *E. crassus* produce the same secondary metabolites, there are some exceptions. For example, several strains do not produce preuplotin (**8**), while other strains do not produce euplotin A (**5**). Some strains produce euplotin C (**7**) only in very low quantities accompanied by trace amounts of preuplotin (**8**). Furthermore, the population SL represents an exception within *E. crassus* being the only strain which does not produce any euplotins at all. From an evolutionary point of view, euplotins are the result of a metabolic pathway, which appears useful in natural selection, regardless of the environmental conditions. This may explain its ubiquity and larger distribution with respect to other morphospecies that share the same habitats, like *E. vannus* and *E. minuta*.

### 3.5. Ecological role and biological activities

At the onset of the investigations, there were two hypotheses regarding the way in which *E. crassus* exchanges chemical information: (i) by the release of substances into the extracellular medium, or (ii) by direct cell-to-cell contacts. Reported studies, carried out *in vivo*, suggested that euplotins are utilized to inhibit populations of competitors via cell-to-cell contact, a good strategy to colonize new habitats or defend settlements [16,27]. Evidence that the toxic effect is a result of euplotins action comes from separate experiments carried out *in vitro* where a non-producing strain of *E. vannus* (TB6) was treated with varying concentrations of pure **7** and a **7**-DIMEB complex at 10 μg/mL. In both cases a 100% reduction on the viable protozoa was observed within one hour. These results confirm that the complexation process does not influence the activity of **7**, while control experiments verified that the effect of dimethylsulfoxide (DMSO) or DIMEB was negligible. At the functional level, euplotin C kills non-producing *Euplotes* cells, whereas at sublethal levels it alters cell cycle, ciliary motility and cell shape [27]. These findings suggest that euplotin C may play an ecological role, representing an adjuvant factor of the multicomponent strategy pursued by *E. crassus* in broadening its niche size.

These results, in conjunction with the fact that many natural products from marine resources show pharmaceutical and medical relevance, prompted the investigation into the potential *in vitro* bioactivity of euplotin C (**7**) against the non-marine pathogenic protozoa *Leishmania major* and *Leishmania infantum*, the opportunistic yeast *Candida albicans* and several prokaryotic microorganisms [40]. The toxicity of **7** against the macrophage-like cell line J774 was used as a mammalian host cell control. DMSO at a non-toxic concentration and DIMEB as a solubilizing agent in water for **7** were also used in the assays. Both *free*-**7** and *complexed*-**7** (*i.e.*, DIMEB-**7** 1:1 inclusion complex) demonstrated good antileishmanicidal activity at 20 μg/mL, causing irreversible fatal damage. After 24 h, LD_50_ values of 4.6 ± 0.5 and 8.1 ± 1.8 μg/mL for *free*-**7** and *complexed*-**7** respectively, were recorded. By contrast, the LD_50_ value was >200 μg/mL for the control J774 cell line. Subsequent studies on the activity of *E. crassus* (a euplotin-producer) and *E. vannus* (a non-producer) towards both *L. major* and *L. infantum* were in agreement with the data from the isolated compound, and found that cellular contact with *E. crassus* SSt22 strain causes irreversibly damage to parasitic cells, while the *E. vannus* TB6 strain does not induce any effect. Although the required amount of euplotin C is too high to be interesting for drug applications, the absence of any cytotoxicity towards the macrophage cells is quite promising.

Further bioactivity results revealed that at high concentrations of >100 μg/mL, **7** showed an inhibitory effect on the yeast *Candida albicans* and on several strains of the bacteria *Streptococcus* and *Burkholderia* spp., whilst at lower concentrations only marginal antimicrobial activity was observed [40]. Interestingly, the *complexed*-**7** form was noted to be more active in longer test times than *free*-**7**, possibly due to the higher chemical stability of the former. An inhibitory effect has also been observed for euplotin C on food vacuole formation and fluid phase endocytosis in *Paramecium primaurelia*, a process that might be mediated by an altered function of cell membranes as well as by modification of the microtubule network [42,43].

The underlying cellular and molecular basis of the effects of euplotin C were recently elucidated using mouse and rat tumor-derived cell lines, followed by the analysis of the nuclear and cytosolic changes usually associated with apoptosis in specific cell lines [29,45]. The apoptotic process is an auto-programmed mode of cell death that is dependent upon energy, as it requires ATP to transmit the death signal from the cytoplasm to the nucleus. It also causes well-defined morphological and biochemical changes in the cell such as cell shrinkage, chromatin condensation, nucleosomal degradation and changes in intracellular cation concentration such as Ca^2+^. The cytotoxicity of **7** was tested on the murine AtT-20 line derived from anterior pituitary tumor cells and the PC12 line originating from a rat chromaffin cell tumor, which expresses properties common to both neurons and neurosecretory cells. It was found that treatment of mouse and rat tumor cells with euplotin C resulted in a dose-dependent cytotoxic effect and in the induction of apoptotic cell death by cellular stress that triggered the activation of the caspase cascade.

Finally, cellular responses to euplotin C in *E. vannus* have recently been investigated by analyzing the cytosolic changes likely to be associated with cytotoxicity and apoptosis, *i.e.*, intracellular Ca^2+^ concentration, organelle components and related caspases [46]. The most important and general outcome of this study is that the chemical signal carried by euplotin is transduced in a rapid impairing of the membrane electrical properties. This causes motility disorders which in turn hinders the ciliate’s ability to escape the external threat. After some minutes the fate of non producers is sealed because the ion channel pumps are completely impaired [43]. These results suggest that euplotin C exerts a marked disruption of homeostatic mechanisms, which play a determinant role in cell-environment interactions.

## 4. Terpenoids from *Euplotes raikovi*

### 4.1. Structures, chemical reactivity and synthesis

**Scheme 5:**
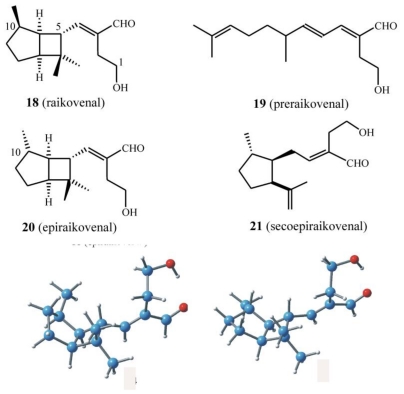
Top: Sesquiterpenoids isolated from cell cultures of *Euplotes raikovi*. Bottom: MM minimized structures of raikovenal **18** [left, ß-Me-C(10)] and epiraikovenal **20** [right, α-Me-C(10)].

While euplotins are built on an unsaturated dioxa-tricyclic system, the structural feature of *E. raikovi* metabolites is the bicyclo[3.2.0]heptane ring system [47]. However, different populations of *E. raikovi* produce C(10)-epimeric metabolites, providing evidence for intra-species variability. In particular, the strain Mor1 collected on the Casablanca coast contains raikovenal (**14**) and its putative biogenetic precursor preraikovenal (**15**), while all other strains so far examined [48] (GA8, SB8, 39W, LPSA5) produce the C(10) epimer of raikovenal (epiraikovenal, **16**) and its seco-analog (secoepiraikovenal, **17**) (Scheme 5).

The chemical and spectroscopic properties of epimeric sesquiterpenoids **18** and **20** are, as expected, quite similar. Although this close structural similarity complicates the de-replication process, the two epimers can easily be distinguished by NMR spectroscopy since the ^13^C resonance of Me-C(10) in **20** is almost 6 ppm downfield with respect to the same carbon resonance in **18**. The opposite trend is observed for the proton resonance of Me-C(10), which results in an upfield shift of 0.05 ppm in **20** with respect to **18**. This turns out to be very useful in the de-replication process, since this signal can easily be detected, even in ^1^H-NMR spectra performed on crude cell extracts.

**Scheme 6:**
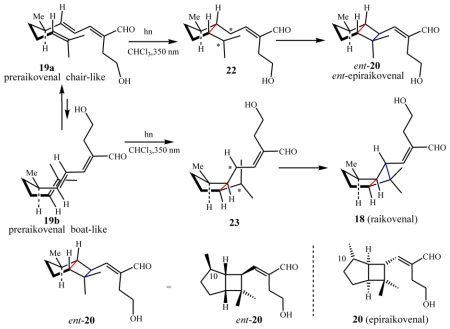
Photochemical conversion of preraikovenal (**19**) into raikovenal (**18**) and *ent*-epiraikovenal (*ent*-**20**).

While the structure elucidation of these metabolites was in progress, interesting investigations began into the photochemical conversions among xenicane diterpenes isolated from a brown alga of the genus *Dictyota* [49,50]. Motivated by the success of these studies, it was hypothesized that the bicyclo ring system embedded in raikovenal could be obtained by direct photo-irradiation of its putative precursor, preraikovenal (**19**) (Scheme 6). Since [2 + 2]-cycloadditions are photochemically allowed processes, it was decided to sacrifice the small amount of the natural isolated compound **19**, in an attempt to obtain an intramolecular photochemical cyclization leading to **18** and/or to **20**. Irradiation at α 350 nm of a chloroform solution of preraikovenal produced raikovenal as a minor product, while the major product had a ^1^H-NMR spectrum that was superimposable to that of epiraikovenal (Scheme 6). According to the proposal [48], the photochemically produced raikovenal **18** could derive from a [2 + 2] concerted reaction (passing through the diradical intermediate **23**) from the boat-like conformation of preraikovenal (**19b**). On the other hand, the photochemically produced epiraikovenal **20** can be explained only by assuming **19** adopts a chair-like conformation (**19a**), and passes through intermediate **22**. However, if one looks at Scheme 6, the expected stereoisomer from the intramolecular addition of the reactive chair-like conformation is not **20**, but its enantiomer ***ent***-**20**. Indeed, it was demonstrated that the major product (epiraikovenal **20**) obtained after purification of the photoaddition product mixture showed a circular dichroic (CD) spectrum opposite to that of natural epiraikovenal **20**, thus indicating the compound to be *ent*-**20**. Although the absolute configuration at the chiral center at C(7) of natural **19** has not yet established, the outcome of this experiment clearly indicates not only that the photoaddition is a strongly stereocontrolled process but also that the inversion of chirality at C(7) is required in order to obtain natural epiraikovenal **20**.

Two independent and different strategies for the total synthesis of racemic **18** and **20** have been reported. The first synthesis described exploits the photochemical methodology in the key step of the bicyclo[3.2.0]heptane ring construction [51]. In the same year (1997), Rosini *et al.* [52], relying on a completely different approach, reported another interesting total synthesis of racemic raikovenal.

### 4.2. Biogenetic considerations

**Scheme 7:**
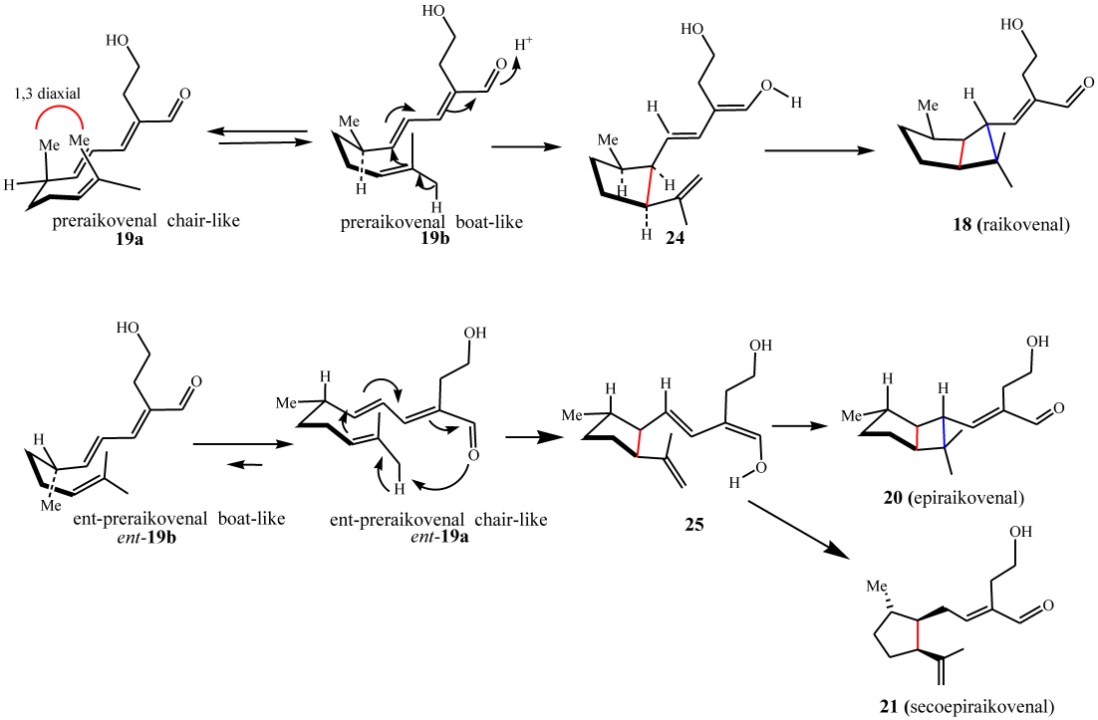
Biogenetic proposal for *Euplotes raikovi* sesquiterpenoids.

According to the results discussed above, if it is assumed that preraikovenal (**19**) is the putative biogenetic precursor of natural raikovenal (**18**), this would suggest that the enzymes involved in the bicyclic ring formation preferentially accommodate and recognize substrate **19** in a boat-like conformation (**19b**). This is supported by MM calculations, which show this conformation to be relatively stable due to the release of the repulsive 1,3-diaxial interaction of the methyl groups in the relevant transition state between preraikovenal and the intermediate **25** (Scheme 7). In order to explain the production of **20** and **21**, an enantiomeric chair-like preraikovenal form, *ent*-**19a**, would be required. Since the photocyclization of preraikovenal (**19**) gave mainly *ent*-**20**, the conformer *ent*-**19a** is proposed herein to be the biogenetic precursor of epiraikovenal (**20**).

### 4.3. Intra-morphospecies phylogenetic analysis

**Figure 5:**
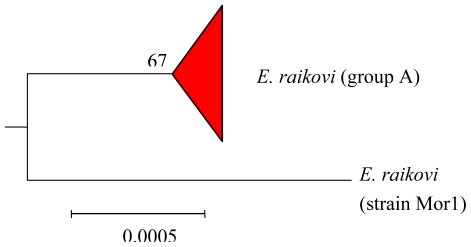
Schematic representation of the phylogenetic tree, based on SSU-rRNA gene sequence comparisons, of different strains of *Euplotes raikovi*. The number at the node is the bootstrap percentage from 1000 replicates. The scale bars correspond to a distance of 5 substitutions per 10000 nucleotide positions.

*Euplotes raikovi* appears as a group with intra-morphospecific heterogeneity, characterized by the synthesis of one or two related terpenes. In particular, the strain Mor1 contains raikovenal and its putative biogenetic precursor preraikovenal, while all other strains so far examined produce the C(10) epimer of raikovenal (epiraikovenal) and its seco-analog (secoepiraikovenal). Considering that one metabolite and its epimer require different biogenetic pathways, it was hypothesized that the production of functional alternatives embedded in the same bicyclo[3.2.0]heptane skeleton is an ecological strategy of adaptation that could also be seen by phylogenetic studies. The phylogenetic tree obtained for *E. raikovi* shows the evolutionary split between the strain Mor1 and the fairly homogeneous group comprised of representatives of other strains with cosmopolitan distribution (Figure 5). This is in fair agreement with the natural product divergence discussed above. As with the euplotins for *E. crassus*, the production of raikovenal/epiraikovenal structures by all strains enables their use as taxonomic markers at the morphospecies level.

### 4.4. Ecological role and biological activities

The terpenoids from *E. raikovi* are less active than euplotins against other ciliates belonging to the genus *Euplotes*. Greater activity was observed [48] against *Litonotus* strains, ciliates with a characteristic predacious life-style which are killed by **18** at concentration as low as 10 μg/mL, and by **19**–**21** at higher concentrations. It is worth to note that euplotin C (**7**) was ineffective against the same predator suggesting the possibility of fine-tuned recognition phenomena in ciliated cells. Biological assays on raikovenal and related molecules demonstrated no killing activity against other ciliates even at concentrations above 20 μg/mL. Two particular features were noted in these experiments. First, epiraikovenal was able to depress the fission rate in *E. vannus* and *E. rariseta* strains. However, this effect does not seem to be specific, as these strains are sensitive to various aldehyde-terpenoids. Second, it appears that certain *E. raikovi* strains use a combination of preraikovenal-raikovenal and epiraikovenal-secoepiraikovenal as a defensive strategy. These compounds appear to be effective only in synergy against predators such as *Litonotus*, accounting for the non-palatability of the strains [48].

## 5. Terpenoids from *Euplotes vannus*

When attention was focused on the secondary metabolites of the morphospecies *Euplotes vannus*, another member of the marine *Euplotes vannus-crassus-minuta* species complex, it soon became apparent that the greater biodiversity of the *E. vannus* morphospecies is mirrored by a greater diversity of secondary metabolites. The number of *E. vannus* strains analyzed so far is fewer than for *E. crassus*, reflecting the lower probability in isolating these organisms from sand samples. However, these analyses suggest that several *E. vannus* subgroups differ in terms of their metabolic productions. This scenario is likely to result from a divergence from a common ancestor and/or intra-morphospecies variability.

### 5.1. Structures, chemical reactivity and synthesis

**Scheme 8:**
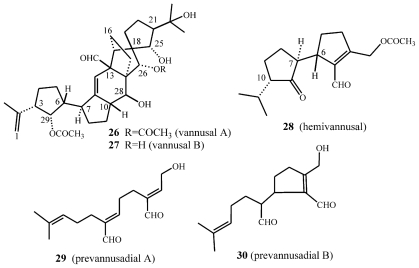
Terpenoids isolated from cell cultures of *Euplotes vannus*.

As summarized in Scheme 8, from tropical strains (Sil21, BUN3) of *E. vannus* morphospecies, two unusual compounds with a novel C30 backbone, named vannusal A (**26**) and vannusal B (**27**) were isolated [53]. On the other hand, investigation of two different strains (TB6 and CM1) led to the isolation of new regular sesquiterpenoids such as hemivannusal (**28**) and prevannusadial A (**29**) and B (**30**) [54].

The structure elucidation of vannusals was extremely challenging [53]. Although the molecular formula suggested mono- and di-acetylated triterpenoid structures, it soon became evident that these were not regular triterpenes deriving from squalene or squalene-oxide internal cyclizations, and in fact cannot be considered triterpenoids at all. There is high structural complexity in the central part of these structures, wherein five- and six-membered rings are interlaced in fused, bridged and spiro forms. A further difficulty arose from the violation of the “isoprenic rule” in these metabolites in the central and more intricate part of the molecules. Natural products chemists’ penchant for this rule is well known since it poses strong constrains on the overall skeleton, thereby minimizing the number of candidate structures compatible with the spectral data. Much assistance in the structure determination was obtained by functional group transformation of **26** into a series of derivatives [53], which ultimately revealed the intricate nature of the vannusal A structure.

**Figure 6:**
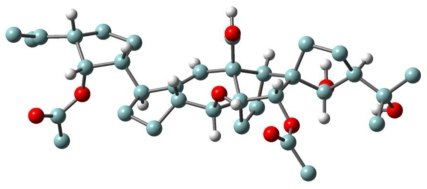
Minimized **s**tructure of vannusal A (**26**). Only -H atoms on chiral centers are represented.

The relative stereochemistry of several chiral centers in the vannusals has recently been revised (Scheme 8 and Figure 6) [54–61]. The stereoselective total synthesis of vannusal A and B (**26** and **27**) carried out by Nicolaou *et al*. confirms the overall structure and main stereochemical architecture of vannusals. In fact, if the relative stereochemistry at C(25) is not considered, the only difference in terms of the relative configurations between the proposed [53] and the revised structures [54,56,60,61] of vannusals derive from the mis-assigned relative stereochemistry at C(6)–C(7). This led to the assignment of the wrong relative stereochemistry of the chiral centers in the “eastern” domain of the molecule with respect to those in “western” one. The brilliant syntheses designed and executed in recent years in the Nicolaou laboratory has finally solved this intricate matter through the total synthesis of eight diastereomeric vannusal B structures [60,61].

Even the structural elucidation of hemivannusal **28** [54] was not a simple task. In particular, the assignment of the relative stereochemistry of its 3 chiral centers, which requires correlation between the stereochemistry of the chiral centers on the rings linked together via a carbon-carbon single bond (C6–C7), around which free rotation is expected to occur. Thus, both the relevant vicinal coupling (^3^J_6,7_) and the dipolar mutual effects (NOE) between the same protons H(6) and H(7) represent averaged values among several C6–C7 rotamers. Assistance in resolving this problem was obtained through an extended conformational search performed within a molecular mechanics approach and refined by *ab initio* quantum chemical calculations.

The minor metabolites of the CM1 strain, prevannusadial A (**29**) and prevannusadial B (**30**), were isolated in very low amounts. Their structures were established [54] by direct comparison of their ^1^H-NMR spectra with those reported in the literature for the “diterpenoid 9” [62] isolated by Paul and Fenical from a Caribbean *Udotea* species (*U. flabellum*) and for petiodial isolated by Fattorusso *et al*. [63] from the Mediterranean *U. petiolata*. Prevannusadial B represents the deprenyl analog of these *Udotea* diterpenoids. This situation has a strong resemblance with that already discussed of the linear sesquiterpenoid preuplotin (**8**), with udoteal isolated from the tropical green alga *U. flabellum* [28].

### 5.2. Biogenetic considerations

**Scheme 9:**
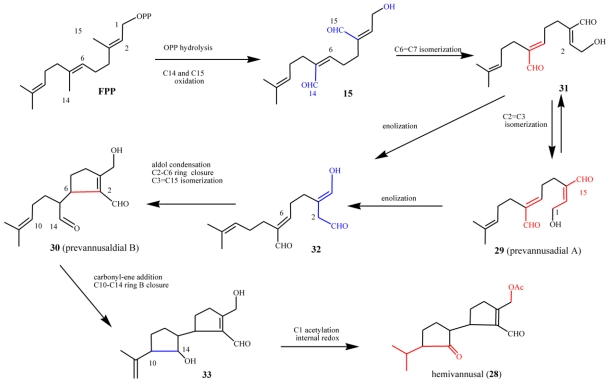
Biogenetic proposal for *Euplotes vannus* sesquiterpenoids.

The possible biosynthetic origin of *E. vannus* metabolites is proposed in Scheme 9 along similar lines to that of the *E. crassus* terpenoids. The biosynthesis of hemivannusal **28** may derive from FPP and prevannusadial A (**29)** precursors through three enzymatic processes. After C1 pyrophosphate hydrolysis, C(14) and C(15) methyl group oxidation would afford the putative intermediate **15** (see also Scheme 4) bearing two α,β-unsaturated aldehyde functions with Z stereochemistry. Following Z→E isomerization of the C6,7 double bond, the intermediate **31** could then lead i) to **29**, after Z→E isomerization of the C2,3 double bond and ii) to the intermediate **32**, after oxidation of the allylic hydroxyl group of **29** and enolization of the C(15) aldehyde group. The intermediate **32** can be converted via intramolecular addition of the activated nucleophilic center C(2) to C(6), an electrophilic center due to its α position in the conjugated unsaturated system, leading to prevannusadial B (**30**). The latter could readily react through an intramolecular C(14)–C(10) carbonyl-ene process affording the bicyclic intermediate **33**. The main metabolite of the strain TB6, hemivannusal (**28**), can be obtained after i) acetylation of the free primary –OH group and ii) internal redox reaction taking place in the saturated cyclopentane ring, a process having precedent in literature reports on carbonyl-ene synthetic procedures [63].

**Scheme 10:**
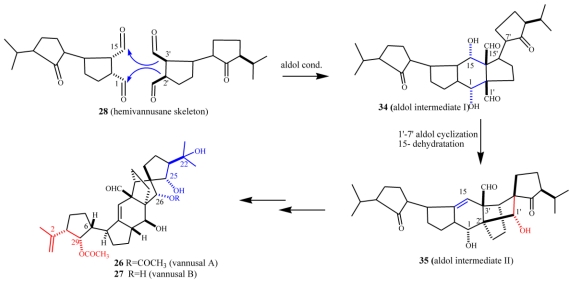
Biogenetic proposal for *Euplotes vannus* C30 terpenoids (vannusals).

As already outlined in the proposed euplotin biosynthesis (Scheme 4), the unusual route involving methyl oxidation and nucleophilic additions seems to be a feature shared by strains belonging to the morphospecies *E. vannus.* Divergence among secondary metabolites of the two species occurs at the level of the putative common intermediate **15**. Furthermore, it relies on the strong preference for acetalization processes in *E. crassus* leading to the highly strained tricyclic skeleton of euplotins, compared to carbonyl-ene reactions in *E. vannus* strains leading to the hemivannusane skeleton. Further evidence of this is given by the C30 terpenoids vannusal A and B (**26** and **27**). Although first thought to arise from a deviation of the squalene pathway to triterpenes, they are now considered as end-products of the nucleophilic condensation of two molecules possessing the regular hemivannusane sesquiterpenoid skeleton (**28**), via the hypothetical aldol-derived intermediates **34** and **35** (Scheme 10). The C30 terpenoids are possibly derived from a concerted condensation of the “peripheral” C1 and C15 electron-poor carbonyl groups of the first hemivannusane intermediate with the “internal” C2′ and C3′ electron-rich centers of the second intermediate. This biosynthetic proposal was exploited by Nicolaou *et al.* in their recent stereoselective total synthesis of the vannusals **26** and **27** [60,61].

### 5.3. Intra-morphospecies phylogenetic analysis

**Figure 7:**
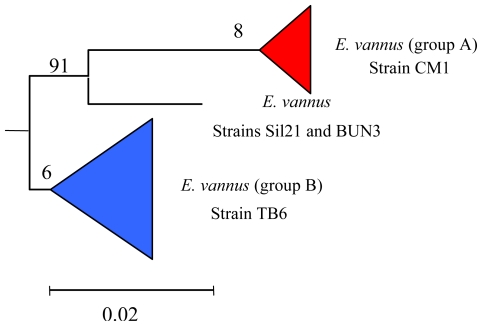
Schematic representation of the phylogenetic tree, based on SSU-rRNA gene sequence comparisons, of different strains of *Euplotes vannus*. The numbers at the nodes are bootstrap percentages from 1000 replicates. The scale bars correspond to a distance of 2 substitutions per 100 nucleotide positions.

In the morphospecies *E. vannus* the chemodiversity of its secondary metabolites mirrors its genetic diversity, as revealed by the phylogenetic analysis. This significant intra-morphospecific variability in the secondary metabolite production is also reflected in the phylogenetic tree. *E. vannus* branches in at least two distinct genetic groups, with each group comprising representatives producing only one of the different classes of secondary metabolites isolated (Figure 7).

Phylogenetic analysis suggests that all the strains examined to-date constitute a monophyletic clade. From a natural products viewpoint this is advantageous. It means that the evolution of *E. vannus* utilizes different secondary molecular characters, not only useful as chemical biomarkers to distinguish different populations of the same morphospecies, but also as important representative of new chemical structures that could be exploited as new bioactive molecules.

## 6. Terpenoids from *Euplotes rariseta*

### 6.1. Structures, chemical reactivity and synthesis

**Scheme 11:**
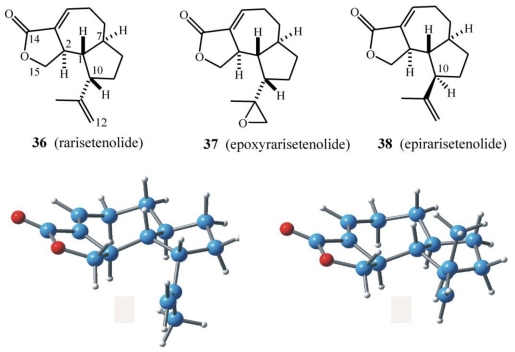
Top: Terpenoids from cell cultures of *Euplotes rariseta.* Bottom: MM minimized structures of raristenolide **36** [left, ß H-C(10)] and epirarisetenolide **38** [right, α H-C(10)].

Analysis of the sesquiterpenoids isolated from *E. rariseta* (Scheme 11) suggests a scenario similar to that already discussed for *E. raikovi.* For example, different strains of the same morphospecies collected in different geographical areas produce two epimeric sesquiterpenoids, rarisetenolide (**36**) (and its epoxide **37**) and the corresponding C(10) epimer, epirarisetenolide (**38**) [65], built on a novel octahydroazulene ring system. So far, only the relative configurations of these terpenoids have been established.

In a series of experiments aimed at elucidating the structures of these terpenoids, the expected mixture of hemiacetal epimers at C(14) was obtained in good yields by DIBAL reduction of the lactone group of **36**. At the same time, it was also demonstrated that the α-ß conjugated lactone could be removed by nucleophilic addition at the unsaturated C(3)=C(4) double bond. Indeed, UV irradiation (α 254 nm) of a methanol solution of rarisetenolide (**36**) in a quartz cuvette at room temperature gave good yields of the corresponding 4-methoxy derivative. Addition of MeOH rather than loss of conjugation under UV irradiation is in accordance with the proposed lactone structures.

As for the C(10) epimeric raikovenals **18**/**20**, the structural similarity between the C(10) epimeric metabolites **36**/**38** complicates the dereplication process. However, the problem is readily solved using NMR spectroscopy. The ^1^H resonance of Me-C(10) (δ_H_ 2.90, dt) in rarisetenolide is 0.5 ppm downfield with respect to the same proton resonance in epirarisetenolide (δ_H_ 2.49, dt), and their vicinal coupling patterns are also different. These signals can easily be detected in ^1^H-NMR spectra, even in crude cell extracts, and thus it is possible to distinguish which one of the two epimers, **36** or **38**, a given strain produces. Until now, a strain of *E. rariseta* that produces both epimers has not yet been found. This is indicative of a selective and/or different enzymatic pool in populations of this ciliate or the use of enantiomeric biogenetic precursors.

### 6.2. Biogenetic considerations

**Scheme 12:**
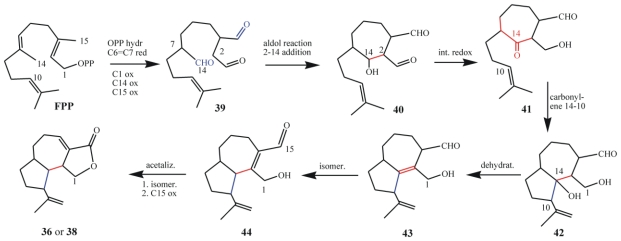
Biogenetic proposal of *Euplotes rariseta* metabolites.

The proposed biosynthesis of rarisetenolide from FPP via the intermediate **44** is shown in Scheme 12. After C1 pyrophosphate hydrolysis of FPP, oxidation of the C(14) and C(15) methyl groups would afford the putative intermediate **39** bearing a saturated aldehyde at what was the C(6)=C(7) double bond. Aldol condensation of this aldehyde with the activated nucleophilic center at C(2), followed by proton tautomerization leads to the intermediate **40**. This could then react through an intramolecular C(14)–C(10) carbonyl-ene process affording the bicyclic intermediate **42**, which upon dehydration produces **43**. This intermediate is proposed to play a pivotal role undergoing acetalization/oxidation processes leading to rarisetenolide-like metabolites through intermediate **44**. Herein, C(14) is thus considered as the electrophilic center for the nucleophilic attack of both C(2) and C(10). If it is considered that FPP methyl groups are rarely involved in intramolecular terpenoid cylizations, the enzymatic processes able to build the octahydroazulene ring system of **36** or **38** appears to be a unique feature of ciliate secondary metabolism. It should be noted however, that the putative linear precursor (*i.e.*, “prerarisetenolide”) has not yet been isolated. The C(6)/(7) hydrogenated analog **39**, the C(14) oxidized form of preraikovenal (**19**), should contain all the structural and stereochemical features required to produce the octahydroazulene ring system of **36** and **38**. Furthermore, it could also give rise to divergent routes towards **36** or **38**, depending on the absolute configuration at its C(7) chiral center. Thus, it is proposed that evolutionary differentiation among *E. rariseta* strains could rely on C(7) enantiomeric FPP oxidized precursors, such as **39**, in a similar way as differentiation among *E. raikovi* could rely on the enantiomeric FPP oxidized precursors **19** and ***ent***-**19**.

### 6.3. Intra-morphospecies phylogenetic analysis

The strains of the morphospecies *E. rariseta* analyzed for the secondary metabolite production have been collected from various coastal sites, including those of northern and southern Australia (strains PD16 and PBH1, respectively), southern Brazil (strain BR1), Canary Islands (strain GRH5), and New Zealand (NZ2) [65]. All of them are found to be producers of non-aldehydic terpenoids, which is quite unusual in the *Euplotes* genus. The three sesquiterpenoids identified, rarisetenolide, epoxyrarisetenolide, and epirarisetenolide, share the same octahydroazulene moiety, which seems to be a convincing chemotaxonomic marker of the morphospecies.

**Figure 8:**
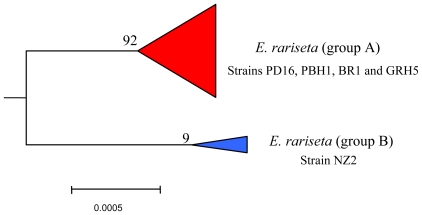
Schematic representation of the phylogenetic tree, based on SSU-rRNA gene sequence comparisons, of different strains of *Euplotes rariseta*. The numbers at the nodes are bootstrap percentages from 1000 replicates. The scale bars correspond to a distance of 5 substitutions per 10000 nucleotide positions.

As with *E. crassus* and *E. vannus*, this morphospecies also shows intra-morphospecific variability in its secondary metabolite production that is reflected in the phylogenetic partition. The four strains of *E. rariseta* (PD16, PBH1, BR1 and GRH5) produce both rarisetenolide and epoxyrarisetenolide. These strains, although collected from geographically distinct locations, are all phylogenetically grouped together in the same clade (Figure 8). It is difficult to believe that this is a coincidence. The production of secondary metabolites is related to enzymes coded by the organism’s genome, their presence reflecting the expression of functional genes, and the inference of a close phylogenetic relationship among the above strains is warranted. In contrast to the other strains, the strain NZ2 gives epirarisetenolide, revealing a subtle variability in secondary metabolism for this ciliated morphospecies. The epimeric relationship of this metabolite with rarisetenolide implies the presence of different metabolic pathways in strain NZ2 with respect to the other strains. This is indicative of genetic differentiation of strain NZ2 as revealed by the phylogenetic tree (Figure 8).

### 6.4. Biological activities

Cytotoxicity of rarisetenolide (**36**) and its congeners (**37** and **38**) against representatives of the marine interstitial ciliate community, comprising *E. rariseta* itself, was detected only at relatively high doses, *i.e.*, >20 μg/mL. The only exception observed was towards the ciliate *Litonotus lamella*, which proved sensitive to **36** at lower doses. It is worth noting that *L. lamella* differs from all other ciliates involved in the bioassays due to its behavior as a predator. It can be proposed that the effectiveness of **36** against *L. lamella* is part of a defensive strategy of *E. rariseta* to avoid predation. Since lower cytotoxicity of the chemically more reactive epoxide **37** with respect to **36** or **38** was found in the ecological tests, interactions with membrane receptors of the *Euplotes* morphospecies might be non-covalent in nature.

## 7. Terpenoids from *Euplotes focardii*

### 7.1. Structures, chemical reactivity and synthesis

**Scheme 13:**
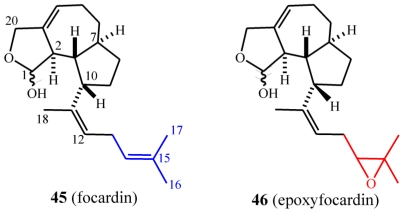
Diterpenoids from cell cultures of *Euplotes focardii.*

Strains assigned to the species *E. focardii* have been found to produce the focardins (Scheme 13) [66], which show much of the structural characters of the rarisetenolide/epirarisetenolide family. From the morphospecies collected from Antarctica (strain TN1) and inter-tropical coastal waters (strains GBS1 and GBS5), a new skeletal class of diterpenoid, epoxyfocardin (**46**), was isolated. Focardin (**42**), which is a possible biogenetic precursor of **46**, was also isolated as a minor component. Focardins constitute the first example of ecologically relevant metabolites from ciliate species that inhabit polar ecosystems.

**Scheme 14:**
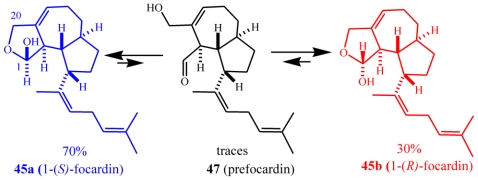
Hemi-acetal equilibration among C(20) epimers of focardin (**45**).

Focardin (**45**) was isolated as a 70:30 mixture of hemiacetals **45a** and **45b**. These equilibrated through the unstable ring-opened, aldehyde intermediate **47** (Scheme 14), to ultimately give epoxyfocardin (**46**) as an 85:15 mixture of its corresponding hemiacetals **46a** and **46b**. Evidence of the equilibrium of the diasteroisomeric hemiacetals came from the analysis ^1^H-NMR spectra of pure **46a** obtained by reversed-phase chromatography of raw extract containing **46** which was superimposable to that obtained before the separation. An unaltered ratio of products clearly suggests equilibrium conditions among the epimers. The absolute configuration of **46a**/**46b** was determined from Mosher’s method [30] through esterification of the hemiacetal hydroxyl group.

### 7.2. Biogenetic considerations

**Scheme 15:**
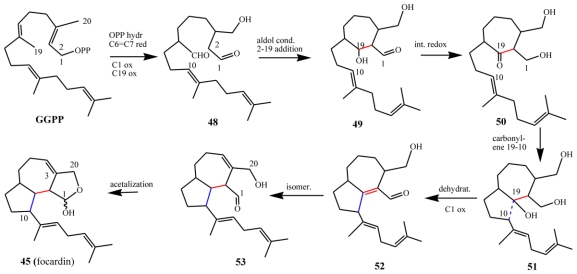
Biogenetic proposal for *Euplotes focardii* metabolites.

The proposed biosynthesis of the *E. focardii* metabolites described here commences from geranylgeraniol pyrophosphate (GGPP) and involves (in the first steps) similar intermediates to those involved in rarisetenolide biosynthesis, albeit with one prenyl unit more (Schemes 12 and 15). Herein, C(19) is playing the same role as C(14) in the raristenolide pathway, but a striking difference between rarisetenolide and focardin biosynthesis is represented by the different oxidation states of C(1) and C(15)/C(20). Whereas in the formation of **36** (or **38**), C(1) is in a lower oxidation state than C(15), in **45** (or **46**) it is in a higher oxidation state than C(20).

### 7.3. Intra-morphospecies phylogenetic analysis

**Figure 9:**
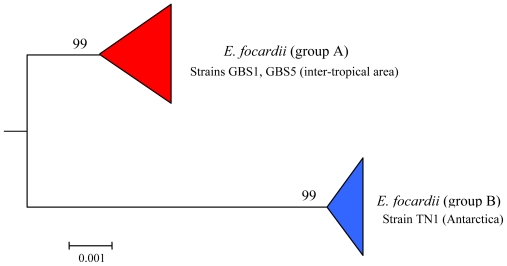
Schematic representation of the phylogenetic tree, based on SSU-rRNA gene sequence comparisons, of different strains of *Euplotes focardii*. The numbers at the nodes are bootstrap percentages from 1000 replicates. The scale bars correspond to a distance of 1 substitution per 1000 nucleotide positions.

By morphological and morphometrical analysis, all Antarctic and inter-tropical populations of *E. focardii* constituted a single cluster of indistinguishable units, thus allowing their attribution to the same morphotype. Nevertheless, within the morphospecies two distinct groups, which corresponded to the two geographical areas of sampling (Antarctic and inter-tropical regions), were detected by phylogenetic analysis (Figure 9). Interestingly, this genetic polymorphism is also reflected by secondary metabolite production. In fact **45** and **46** can be isolated in higher yields from the inter-tropical strains GBS1 and GBS5, whereas the Antarctic TN1 strain produces them only in low amounts (Figure 9). This observation suggests that competition for exploitable niches and food varies with latitudes, thus the survivor strategies are also expected to vary. In particular in the inter-tropical area the metabolite expression would be expected to be higher, since *E. focardii* inter-tropical populations must cope with higher species competition than the Antarctic strains of the same morphospecies.

### 7.4. Biological activities

The paucity of suitable test organisms prevented the investigation of the ecological role of focardin and epoxyfocardin, but the high bioactivity of the latter against predacious ciliates such as *L. lamella* and *L. cygnus* suggests a defensive role for these substances. This would indicate that ciliates are in strong competition even in Antarctica. Of the two diterpenoids, focardin (**45**) showed the least cytotoxic activity. *E. focardii* showed a significant cytotoxic activity *vs*. various ciliate morphospecies, including congeneric species of the ciliate *Litonotus*, a notorious predator of *Euplotes* specimens. Due to difficulties in accomplishing long lasting experiments using psychrophilic ciliate strains (growing at temperatures close to water-freezing), after 24 h of *Litonotu*s predaction activity, the disappearance of *Euplotes* representatives of different morphospecies in the sample community was significantly larger than that observed when the sample ciliate community included specimens of *E. focardi*. Toxic activity counteracting predation by *Litonotus* may be guessed for the substances produced by *E. focardii*. Focardin’s low bioactivity and low abundance in *E. focardii* infers a minor role for this metabolite in nature.

## 8. Functional Role and Phylogenetic Significance

The modern construction of phylogenetic trees and evolutionary events concerning species is based on molecules that are found in all organisms, such as ribosomal RNA (rRNA) and highly conserved proteins, or polymorphic enzymes for population structure and dynamics. Some species, however, compete for space and resources, or defend themselves from predators, largely due to their secondary metabolites, which are potentially their best taxonomic and phylogenetic markers [67].

It is well known that terpenes act as defensive substances for a variety of marine organisms including algae, sponges, corals, mollusks and fish [71]. For example, caulerpenyne is an acetylenic sequiterpene found in certain species of green algae, which is rapidly converted into highly reactive 1,4 dialdehydes by esterase enzymes that are activated upon wounding [69]. Chemical defense is also typical in the poorly defended, shell-less opisthobranch mollusks, which accumulate terpenes both from their diet and from *de novo* biosynthesis [70].

As described recently [71], biosynthetic pathways leading to secondary metabolites may have evolved to favor molecular diversity, although the explanation on how the evolution has followed its course is a matter of debate. From the point of view of the “target-based” model of natural product evolution [72,74], certain secondary metabolites could confer a survival benefit to an organism through their ability to specifically bind to cellular targets in competing organisms. This belief, however, relies on the assumption that natural product/receptor interactions are comparable to enzyme/substrate interactions [72]. From the point of view of the “diversity-based” model (originally named the “screening” model [73,75]), evolution would favor organisms that “could generate and retain chemical diversity at low cost”. Here the assumption is that organisms producing a wider chemical diversity should have greater chances of producing metabolites with potent biological activity [75]. Herein, almost two decades of comparative analysis of the secondary metabolism and phylogenesis of marine ciliates of the family Euplotidae from temperate and tropical marine environments is described. The synergistic contribution of expertise coming from biological (microbiology, molecular biology and ecology) and chemical (analytical and synthetic organic chemistry) disciplines has created the opportunity to propose a global picture of the ciliate protistan world to-date.

Marine interstitial ciliates belonging to the genus *Euplotes* have emerged as a rich source of new terpenoids bearing various functionalities. Furthermore, these terpenoids display activities against other ciliates, competing for space and resources, and also possess interesting biological properties such as apoptotic activity towards mouse and rat tumor cell lines [29,45]. Generally, the lipophilic nature of these compounds indicates that their principal targets are likely to be cell membranes, wherein they could play a role in chemioosmotic control [46]. Moreover, there is evidence from bioassays that terpenoids are non-covalently bound to the ciliate’s external membrane.

**Scheme 16:**
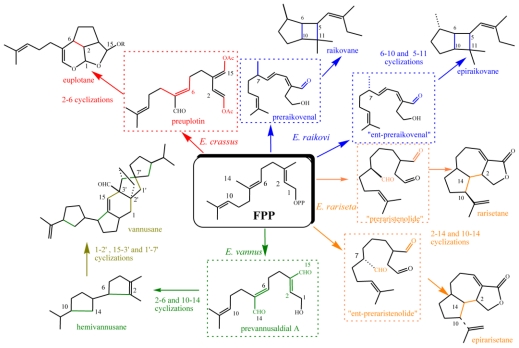
Metabolic routes towards terpenoids produced by marine ciliates of the genus *Euplotes*.

From an evolutionary point of view, the structural chemo-diversity among ciliate metabolites is not unexpected because it reflects the biological imperative to maximize biodiversity starting with both a minimal substrate pool and minimal genetic alterations [76]. It also provides evidence that the biodiversity and adaptive ability of marine ciliates is higher than what was previously thought. From a structural point of view, many of the ciliate secondary metabolites isolated so far are regular, often polycyclic terpenoids, which are proposed to originate from the biogenetic precursors FPP or GGP through the involvement of species-specific enzymes. In particular, four different sesquiterpenoid skeletons have been isolated and demonstrated to be species-specific: *i.e.*, euplotane (*E. crassus*), raikovane (*E raikovi*), rarisetane (*E. rariseta*) and hemivannusane (*E. vannus*) (Scheme 16). The latter is thought to be the biogenetic precursor of the unusual C30 vannusane metabolites. The putative linear precursors to three of the above (preuplotin, preraikovenal and prevannusadial A), deriving from methyl oxidations of FPP along with C(6)=C(7) double bond isomerizations, have been isolated from strains of the corresponding morphospecies (*E. crassus*, *E. raikovi* and *E. vannus*, respectively). On the other hand, the proposed linear precursor of rarisetenolide (“preraristenolide”) has never been found in any strain of *E. rariseta*. The unifying picture of the secondary metabolites in marine ciliates depicted in Scheme 16 assumes that epimeric products **18/20** observed in *E. raikovi*, and **36/38** in *E. rariseta*, should derive from enantiomeric forms of the corresponding linear precursors. It has also been demonstrated that another diterpenoid structure, focardane, is a chemotaxonomic marker of the morphospecies *E. focardii*.

**Figure 10:**
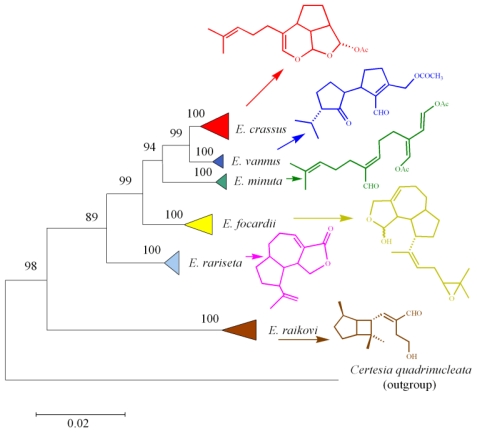
Schematic representation of the phylogenetic tree, based on SSU-rRNA gene sequence comparisons, of different species of *Euplotes* and their main terpenoid secondary metabolites isolated to-date. The numbers at the nodes are the bootstrap percentage from 1000 replicates. The scale bars correspond to a distance of 2 substitutions per 100 nucleotide positions.

To gain insight into the phylogenetic relationships among *Euplotes* species, SSU-rRNA gene sequences of representatives belonging to the different marine species were determined and aligned in order to compare molecular phylogenesis with secondary metabolite production. The results obtained clearly define not only the relationships among the different populations of the same morphospecies (intraspecific phylogenetic trees) as already discussed in previous sections (see Figures 4, 5, 7–10), but also the relationships among different *Euplotes* morphospecies (interspecific phylogenetic tree). The latter indicates the monophyly of the taxon *Euplotes*, characterized by the following particular features: (1) *E. raikovi* branches basal to the other species; (2) *E. crassus*, *E. vannus*, and *E. minuta* form a stable group positioned at the apex of the phylogenetic tree; (3) *E. focardii* is the sister species of the previous group; (4) *E. rariseta* occupies an intermediate position and is more phylogenetically related to the clade *E. crassus* - *E. vannus* - *E. minuta* - *E. focardii* than to *E. raikovi* species (Figure 10).

The production of secondary metabolites at the morphospecies level is consistent with the results described above. Three different compounds, the euplotins A–C and their precursor preuplotin, have been isolated from *E. crassus*. The other two members of the *E. vannus-crassus-minuta* complex produce only small amounts of preuplotin (*E. minuta*) or are characterized by the complete absence of euplotins (*E. vannus*). This latter species is instead characterized by the production of a variety of compounds, with different populations of *E. vannus* giving regular sesquiterpenes such as hemivannusal, prevannusadial A, and prevannusadial B, while others give dimeric sesquiterpenes such as vannusal A and vannusal B, and others appear not to produce any terpenoid secondary metabolites. These results suggest (Scheme 16 and Figure 10) that *E. crassus* is a species that has developed a homogeneous set of secondary molecular characters, and evolution of this species is destined either to maintain all these characters (euplotins A–C) or to lose all of them. In contrast, the evolution of *E. vannus* utilizes different sets of secondary molecular characters to distinguish different populations.

From an evolutionary perspective, it could be said that genes implied in the secondary metabolism of the TB6 strain (*E. vannus*) have evolved from divergence of ancestral homologous genes of the CM1 strain, since the latter are (and were) not able to encode enzymes for the biosynthesis (Figures 5 and 6) of the bicyclic terpenoid hemivannusal from prevannusadial A. On the other hand, strains Sil21 and BUN3 should have the most diverse genes since they are able to carry out the reaction even further than hemivannusal, affording “dimeric” C30 terpenoids such as vannusal A. Of course, it also possible that it is not new genes, which have arisen by gene duplication followed by divergence, but an increased expression of genes encoding new enzymes and, hence, new chemical processes which are not expressed in the ancestors.

Some morphospecies appear to be much more homogeneous than others (e.g., *E. raikovi*), suggesting different evolutionary strategies. This result must be interpreted according to the recent reported evidence [39], which shows that within ciliate morphospecies, there is sufficient isolation to allow separation of genomes without morphological differentiation. Moreover, the high number of strains genetically analyzed for each morphospecies indicates the existence of an internal genetic variability that undermines the general assumption that morphospecies equate to an evolutionary unit. Interestingly, these genetic intra-morphospecific groups have their exact counterpart in the outcome of the chemotaxonomic approach. This analysis shows that different strains belonging to the same morphospecies but grouped in different genetic clades, are characterized, both qualitatively and quantitatively, by a different profile of secondary metabolites.

## 9. Conclusions

Unicellular ciliated protists represent an important component of the grazing marine food web, thus playing a key, although scarcely investigated, role in the marine ecosystem. From the structural chemist’s point of view the most fascinating aspect of this trip into the world of ciliates is represented by the high skeletal chemical diversity found in this phylum. Using sesquiterpenoids as an example, the isolation of four new C15 skeleta was unexpected at the beginning of the study, since more than a century of research had been dedicated not only to the identification of new metabolites but also to explain the mechanism of their biosynthesis. Although experimental biogenetic investigations on the origin of ciliate secondary metabolites have not yet been carried out, it is quite clear from the isolated metabolites that marine ciliates have a unique enzymatic system able to deviate from the usual course of the linear terpenoid precursors. Typically, sesquiterpene synthases (cyclases) catalyze the cyclization of FPP into any one of the 300 known hydrocarbon skeletons. This involves ionization of the FPP pyrophosphate leaving group, followed by the generation of a reactive allylic carbocation and subsequent C-C bond formation (along with alkyl shifts), leading to a terminal carbocation that is finally quenched by a base [77]. The analysis of the sesquiterpenoid skeleta elucidated from marine ciliates so far strongly suggests that the methyl groups of FPP are oxidized in the early steps of the biosynthetic route affording the novel linear precursors. Thereafter, the corresponding aldehyde carbonyl groups are extensively used as electrophilic centers in subsequent reactions. Thus, in the biosynthetic proposals herein, the oxidations which occur in the early steps may predispose the linear precursors to follow a particular path towards the novel end-products observed. The challenges for the future will be to substantiate this view with experimental findings, to isolate and characterize the terpene cyclases involved and to exploit the great advancements of molecular biologists towards mapping of the marine ciliate genome. The powerful tools of molecular biology coupled with recent advances in analytical chemistry will enable future investigation into terpene production from marine ciliates “manipulated” by genetic knockouts or by the use of low-molecular-weight elicitors.

Finally, it is often reported that secondary metabolite production could be mediated by prokaryotic (bacteria) endosymbionts harboured by eukaryotic microorganisms. Actually, ciliate cells represent a suitable niche for endosymbiotic colonizers, mainly of prokaryotic nature. Furthermore, specific bacterial endosymbionts characterize several *Euplotes* taxa and are known to infect all the members of the comprised populations. Treatment with antibiotic drugs (e.g., penicillin) has demonstrated that specific features of the eukaryotic guests are bestowed on the prokaryotic hosts. *Euplotes vannus* intra-population variability allowed us to isolate both the significantly infected strain TB6 and the endosymbiont-free strain Male5 [54]. According to the phylogenetic analysis based on 18S rRNA, ITS1, ITS2 (nuclear), and 16S rRNA (mitochondrial) gene sequences, Male5 has been demonstrated to be genetically identical to the TB6 strain. The HPLC-MS analysis of an organic extract obtained from a small-scale cell culture of this strain (*E. vannus*, Male5) clearly indicated the presence of the same major secondary metabolite (hemivannusal (**22**)) in almost the same amount as that produced by the *E. vannus* TB6 infected strain. This result strongly suggests that **22**, but reasonably also all the metabolites so far isolated from marine ciliates belonging to the genus *Euplotes*, are “true ciliate metabolites” rather than products of the harbored prokaryotic endosymbionts.
